# Explaining retention of healthcare workers in Tanzania: moving on, coming to ‘look, see and go’, or stay?

**DOI:** 10.1186/s12960-016-0098-7

**Published:** 2016-01-19

**Authors:** Aloisia Shemdoe, Godfrey Mbaruku, Angel Dillip, Susan Bradley, JeJe William, Deborah Wason, Zoe Jane-Lara Hildon

**Affiliations:** Ifakara Health Institute, Kiko Avenue, Plot 463, P.O. Box 78373, Mikocheni, Dar es Salaam, Tanzania; School of Health Sciences, City University London, 1 Myddelton Street, London, EC1R 1UW England; Oxford Policy Management, GEPF House, Plot No 37, New Bagamoyo Road, P.O Box 71166, Dar es Salaam, Tanzania; Department of Public Health, NHS Ayrshire & Arran, Dalmellington Rd, Ayr, KA6 6AB Scotland; Saw Swee Hock School of Public Health, National University of Singapore, Tahir Foundation Building, MD1, Science Drive 2, 117549 Singapore, Singapore; London School of Hygiene and Tropical Medicine, Faculty of Public Health & Policy, Department of Global Health and Development, Keppel Street, London, WC1E 7HT England

**Keywords:** Mid-level cadres, Managers, Staff shortages, Long- and short-term retention, Tanzania, Multiple and qualitative methods

## Abstract

**Background:**

In the United Republic of Tanzania, as in many regions of Sub-Saharan Africa, staff shortages in the healthcare system are a persistent problem, particularly in rural areas. To explore staff shortages and ways of keeping workers in post, we ask, (a) Which cadres are most problematic to recruit and keep in post? (b) How and for what related reasons do health workers leave? (c) What critical incidents do those who stay face? (d) And why do they stay and cope?

**Methods:**

This is a multi-method paper based on analysis of data collected as part of a cross-sectional health facility study supporting maternal and reproductive health services in the United Republic of Tanzania. Qualitative data were generated through semi-structured interviews with Council Health Management Teams, and Critical Incident Technique interviews with mid-level cadres. Complementary quantitative survey data were collected from district health officials, which are used to support the qualitative themes.

**Results:**

Mid-level cadres were problematic to retain and caused significant disruptions to continuity of care when they left. Shortage of highly skilled workers is not only a rural issue but also a national one. Staff were categorised into a clear typology. Staff who left soon after arrival and are described by ‘Look, See and Go’; ‘Movers On’ were those who left due to family commitments or because they were pushed to go. The remaining staff were ‘Stayers’. Reasons for wanting to leave included perceptions of personal safety, feeling patient outcomes were compromised by poor care or as a result of perceived failed promises. Staying and coping with unsatisfactory conditions was often about being settled into a community, rather than into the post.

**Conclusions:**

The Human Resources for Health system in the United Republic of Tanzania appears to lack transparency. A centralised monitoring system could help to avoid early departures, misallocation of training, and other incentives. The system should match workers’ profiles to the most suitable post for them and track their progress and rewards; training managers and holding them accountable. In addition, priority should be given to workplace safety, late night staff transport, modernised and secure compound housing, and involving the community in reforming health services culture and practices.

## Background

Worldwide, the geographical distribution of health workers is skewed towards urban and wealthier areas. This pattern is found in nearly every country in the world, regardless of the level of economic development and health system organization, but the problem is especially acute in developing countries [1]. There are multiple factors influencing a health worker’s decision to relocate, stay or leave a post in rural or remote areas. Such complex and interconnected factors are linked to a health professional’s characteristics and preferences relating to health systems organization and to the wider social, political and economic environment [2].

In the United Republic of Tanzania, in rural areas where the majority of the population continues to reside, problems of recruiting and retaining health staff are most pronounced [3]. The recruitment and retention debate has tended to focus on absolute numbers of health workers, rather than exploring the dynamics, such as reason for of geographic differences, and health worker flows within (transition/intrasectoral mobility) in and out of (exit/intersectoral mobility/migration) the health workforce [4]. Failure to retain existing staff incurs additional costs to the health system, including training new staff, recruiting replacements and overtime/locum costs to cover the ensuing staff shortages [5]. It also has a detrimental impact on the health workforce team and its skill mix [6]. In the Tanzanian context, distributional inequalities in health worker numbers are mirrored by large skill mix inequalities, where districts with fewer human resources have an even lower share of the highly skilled cadres [7].

Unsurprisingly then, the health workforce in the United Republic of Tanzania has been documented to be very unevenly distributed between rural and urban districts [8]. A recent country survey (SARA) [9] found that 69 % of all health professionals worked on urban facilities. This is despite the fact that roughly 70 % of the population lives in rural areas. It is noted that the health workforce challenges in the country’s public institutions are significantly related to poor working conditions, lack of resources and equipment. This situation drives some staff, especially highly trained ones, to seek private or third sector employment or to move outside the country. Those who remain working in the country can become greatly demoralized [10].

The current analysis draws on what is already well known and striven for in the Tanzanian context. In sum, quantitative studies demonstrate that problems persist and that the difficulties in the United Republic of Tanzania surpass those of many surrounding African nations. It appears that the situation has remained stagnant and slow to change. Qualitative studies have shown that infrastructure limitations and poor morale are persistent issues. It is hoped that fully elucidating qualitative questions, or the ‘whys’ and ‘hows’ [11], of staff shortages will give a fuller picture of what is being experienced on the ground, to help better inform Human Resources for Health policy.

Our aim is to appraise the contexts and processes that drive staff shortages in the United Republic of Tanzania, including problems with recruitment procedures, but focusing mainly on explaining retention shortfalls. Our research contrasts clinical Health Care workers and Management perspectives. The objectives and related research questions are as follows:

1. To explore staff shortages and their reasons
  - Which cadres are most problematic to recruit and keep in post?
  - How and for what related reasons do health workers leave?
    *(Questions a & b are based on views from district health officials and members of the Council Health Management Teams, CHMTs)*
  - What critical tipping points or stressors that push workers to want to leave their jobs, do those who stay face?
2. To explore retention and positive coping
  (d) Why do staff stay and cope?
  *(Questions c & d are based on interviews with mid-level providers, MLP, of emergency obstetric services)*

## Methods

### Study design

Data for the current analyses were collected as part of a mixed-methods cross-sectional study that took place in 2008 in the United Republic of Tanzania. The quantitative component reported herein reports descriptive statistics on Human Resources for Health (HRH) level information by district (*n* = 43) drawing on information provided by district health officials.

**Fig. 1:**
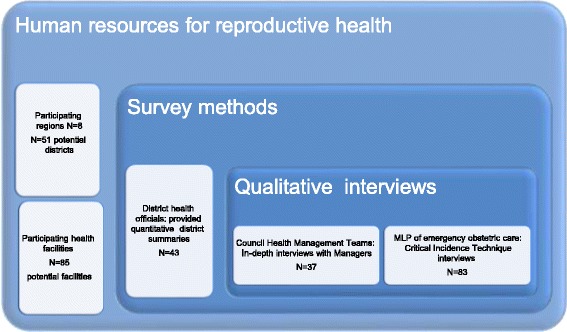
Using multiple methods, complementary qualitative data collection and a survey

The qualitative counterpart includes semi-structured interviews with members of the CHMTs (*n* = 37) on their perspectives of staffing-related issues. We also employed the Critical Incident Technique (CIT) [12], a semi-structured interview with healthcare workers (*n* = 83) that focused on tipping points at work. The CIT is a flexible tool to identify key turning points and their practical implications [13]. This study used the CIT to identify moments that had occurred in the previous 3 months that had made participants seriously consider leaving their job, as a hook to help them talk about why healthcare workers decided to stay and how they cope. Therefore, interviewers aimed to elicit accounts of critical moments, such as the proverbial ‘*straw that broke the camel’s back*’, to explore reasons why health workers stay or leave their work places. Data collection is summarised in Fig. 1.

### Sampling

Sampling was based on the eight United Republic of Tanzania zones. One region from each zone was selected randomly through a multi-stage sampling technique, which brought up eight regions: Dodoma, Pwani, Mwanza, Tanga, Mbeya, Iringa, Tabora and Mtwara. All hospitals and health centres in 51 potential districts from each region were invited to participate. A survey summarising the district’s staffing issues was completed in 43 of the 51 districts, representing 84 % of the potential districts. These comprised both peri/urban (*n* = 9) and rural (*n* = 34) districts.

CHMT members were purposively sampled because their day-to-day role includes managerial responsibilities. We wanted to select a sufficient sample size to provide a comprehensive overview of CHMT perceptions on human resource issues, so researchers were asked to interview at least two CHMT personnel in two districts in each of eight selected regions in the United Republic of Tanzania. Teams tried to obtain the CHMT interviews in the first district visited in each region. If they were unable to secure at least two interviews they moved on to the next district and tried again. They continued in this fashion until they had secured the required quota of interviews. Data were only included from districts where at least two of the key members of the C/DHMT were available to be interviewed at the time of data collection.

A total of 37 CHMT members were interviewed and were District Health Secretaries (*n* = 13), Reproductive and Child Health Coordinators (*n* = 13), District Medical Officers (*n* = 10) and one District Nursing Officer (*n* = 1). The majority of these interviewees were females (*n* = 22) while the rest were males (*n* = 15).

All of the potential facilities in these districts were approached and asked to participate in the CIT interviews, with 83 MLPs working in emergency obstetric care agreeing to take part; these were purposively sampled from a larger ‘Health System Strengthening for Equity’ (HSSE) survey (*n* = 825). Less than half of the interviewees were nurse officers/nurse midwives (*n* = 20) and lower assistant cadres (*n* = 20), while assistant medical officers represented the rest (*n* = 43). The majority (about 75 %) of participants were women.

### Data collection

The fieldwork teams were well trained and comprised of a balance of male and female interviewers, the majority of whom held Bachelor degrees. Interview guides were used to collect data from CHMT members, while the CIT was used to elicit qualitative information from MLPs. Interviews took place in private offices and lasted between one and one and half hours. The survey data was collected face-to-face. All interviews were digitally recorded, transcribed verbatim and supplemented by written notes. Kiswahili transcripts were later translated into English by researchers fluent in both languages. An accuracy and quality check of transcripts was ensured before data analysis.

Permission from heads of each health facility was sought before the interviews were conducted. All surveys and interviews were conducted in *Kiswahili*, the Tanzanian national language. The objectives of the study were explained and respondents were assured of confidentiality of the information provided. All data and records were anonymised from the outset with a unique identity number. Informed, signed consent was obtained from every respondent.

### Data analysis

Quantitative district level data was descriptively analysed, considering statistical differences in rural vs. urban settings, where appropriate (Fishers Exact test), in order to set the context and to triangulate with qualitative data. Thematic analysis [14] of qualitative data was used to identify the explanatory themes in response to our research questions; these themes are *reported in italics*. Attention was given to reporting both majority and minority voices. All analyses were ordered by rural and peri/urban settings. Peri-urban settings have been subsumed into the urban category, since these staffing and resource allocation issues are aligned to urban counterparts.

### Ethical clearance

Ethical approval was granted by the Medical Research Coordinating Committee (MRCC) of the National Institute for Medical Research (NIMR) in the United Republic of Tanzania, and by the Global Health Ethics Committee, Trinity College, Dublin, and the Institutional Review Board of Columbia University, New York.

## Results

### 1. Exploring staff shortages and their reasons

- Which cadres are most problematic to recruit and keep in post?

The survey with district health officials showed, as expected, that recruitment and retention were more often perceived as a problem in rural districts (56 %) compared to urban settings (33 %), Table 1. However, in terms of cadres, Table 2, medical officers and doctors were seen equally to be in shortage (56 %) in both settings, while the least qualified grades, such as medical attendants and Mother and Child Aides, were not perceived as problematic human resources to employ and retain.

**Table 1:** Reports from district health officials on recruitment, retention or both as problematic in their district

Problematic in your district?	Setting *n* (%)		Overall *n* = 43
Totals	Rural *n* = 34	Urban *n* = 9	
Recruitment	9 (26)	2 (22)	11 (26)
Retention	6 (18)	3 (33)	9 (21)
Both (recruitment and retention)	19 (56)	3 (33)	22 (51)
Missing	0 (0)	1 (11)	1 (2)
Total	34 (100)	9 (100)	43 (100)

**Table 2:** Hardest cadres to recruit and retain in their urban or rural district

Hard to recruit and retain?	Setting *n* (%)		Overall *n* = 43
Totals	Rural *n* = 34	Urban *n* = 9	
Medical officer/doctor	19 (56)	5 (56)	24 (56)
Assistant Medical Officer	1 (3)	0 (0)	1 (2)
Clinical officer	6 (18)	1 (11)	7 (16)
Medical attendants	0 (0)	0 (0)	0 (0)
Registered nurse/nurse midwife	1 (3)	3 (33)	4 (9)
Enrolled nurse	0 (0)	0 (0)	0 (0)
MCH Aides	0 (0)	0 (0)	0 (0)
Dentist	1 (3)	0 (0)	1 (2)
Laboratory Technician	1 (3)	0 (0)	1 (2)
Pharmacist	2 (6)	0 (0)	2 (5)
Other	3 (9)	0 (0)	3 (7)
	34 (100)	9 (100)	43 (100)

When district health officials were asked to rank the most needed cadres and those that were hardest to retain, most managers in rural and urban settings stated that the most wanted cadres were as follows: medical doctors, followed by laboratory technicians, clinical officers, midwives, then enrolled/registered nurses. Overall, taking into account CHMTs views too, it appeared that *the perception of retention of higher cadres had shifted from being seen as largely a rural problem to a national one*, as described in urban settings: ‘mostly the doctors [hardest to find and keep] and the reason is doctors have big job market, one can be employed in other good towns […] others are opting to leave the country’. (CHMT, urban). In rural ones, the higher or female cadres were seen to struggle most with the lack of infrastructure, explaining why some came or stayed: ‘because for the doctors and their nurses who are there, they are provided with good modern houses’. (CHMT, rural).

Moreover, it was the rural CHMTs that spoke most about their multiple retention frustrations and a particular demand for cadres *with specialist skill sets, such as anaesthetists and pharmacists*. These managers also spoke specifically about *the disruptions to continuity of care caused by losing nurses and nurse-midwives, or other such MLPs*: ‘…enrolled nurses, the majority of them are leaving, these are the ones who were called nurses midwives before’. (CHMT, rural). *Losing MLPs was seen as especially disruptive, since they are central to ensuring proper follow-up to mother and child*: ‘… it is important for them to remain in the station because of the nature of reproductive and child health’. (CHMT, rural).

Explaining the poor retention of nurses or medical officers *was the very nature of their (mid-level) career stage and the likelihood of these staff leaving to retrain, to become highly or specialist skilled*. As one manager put it: ‘Cadres like nursing officers or midwives, even pharmacists, to get them is difficult and to retain them is also hard, if she goes for studies then often she does not come back’. (CHMT, rural). In addition, *the lack of specialist cadres was described as perpetuated by few trainees actually succeeding in their studies:* ‘Also another thing is about physiotherapists; that is a real problem because they are not available and very few are taking the course. It is also difficult to convince people to take the course; anyway the majority don’t end up qualifying’. (CHMT, rural).

**Fig. 2:**
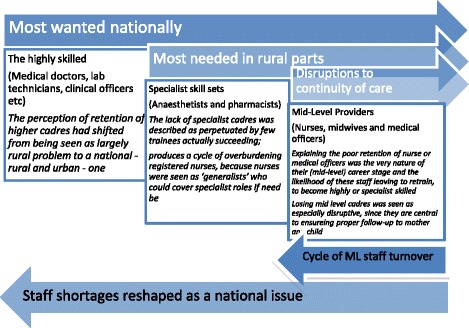
Description of problematic cadres to recruit and keep in post in the United Republic of Tanzania

Concurrently, *the lack of specialist staff produced a cycle of overburdening registered nurses, because nurses were seen as ‘generalists’ who could cover specialist roles if need be*. As noted by one manager: ‘Up until now we don’t have a district pharmacist, there is a nurse who is acting as a pharmacist and she has been there for many years so she has experience and we are grateful to have her…We have tried to employ pharmacists but they do not come; so we have sent one of our workers to be trained on Bachelor of pharmacy, but this will take time’. (CHMT, rural). These relationships are summarised in Fig. 2.

b) How and for what related reasons do health workers leave?

**Fig. 3:**
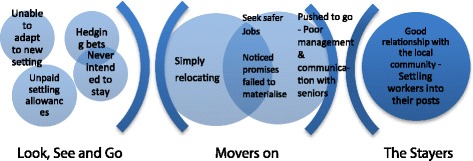
Typology of leavers and stayers

The ways that health workers tended to leave their posts can be classified into two main types. The first type were those who left after a very short term, a phenomenon characterised as *Look, See and Go*, in which healthcare workers left their posts in under 3 months, often leaving within the first few weeks. The second type of leavers were *Movers On*, who were those settled in post for some extended period of time, but become ready to move on. The remaining workers are those who did not leave their post, who can be described as *The Stayers.* The underpinnings of this taxonomy are narrated below, and their core supporting themes summarised in Fig. 3.

*Look, See and Go* leavers were described by managers as those who arrive at duty stations and shortly afterwards are gone. Some ask for a change of work post, while others leave without informing the authority responsible: ‘…they just disappear, you cannot see him/her again’. (CHMT, urban). Although not unheard of in urban parts, this tended to happen mostly in the rural postings. According to one interviewee: ‘Most of them are girls, a girl comes to your office, frustrated and start crying; then they just leave’. (CHMT, rural).

The main explanation for this was that workers were just *unable to adapt to the new settings*. Health workers who were previously urban dwellers were particularly unsettled, confronted with poor mobile communication and transport, lack of safe water and electricity, fear and superstition from resident locals. As this manager explains regarding nursing staff: ‘When they come, they expect to find the house with all necessities like electricity, but when she arrives and informed that she will be taken to [name] dispensary in [name] village, 127 km from municipal where water is also not clean, they refuse to remain and decide to go elsewhere’. (CHMT, rural).

Manager also stressed that some people tended to offset their chances, *hedging their bets* based on several applications, to get a preferred posting, as this CHMT respondent explains: ‘they get both postings and they then choose where to go, looking at [district name] means they might be allocated to remote villages, if this happens they can move as quickly as possible to the urban facilities if this subsequently comes up [even if in a different district]’. (CHMT, rural). Often, candidates may have chosen a district and be hoping for an urban posting, but end up posted in the rural rather than more urban centres.

There was a suspicion that sometimes workers initially move, collect resettlement allowances, *although they may never have intended to stay*, since in these cases: ‘you find they had [deliberately] applied at different places’. On the other hand, it was also described that *unpaid settling in allowances* or subsistence for the newly employed meant that the new workers had ‘no money to start with’, and left shortly after arrival.

As for the *Movers On*, these more settled workers’ leaving patterns crossed two spectrums: from *simply relocating*, for instance to join new spouses or for a better opportunity, to being *pushed to go* due to a critical incident, such as a fallout with a colleague or feeling at risk. In some cases, these explanations are blurred and overlapped. The most valued workers were seen to leave to join more prestigious organisations such as NGOs. *Movers On* were often described as wanting to take on ‘cleaner’ clerical roles, basically to seek *safer jobs*. These were powerfully described as jobs, which ‘did not include any contact with blood’.

Some *Movers On* were said to recount wanting to start a business, such as a retail pharmacy, or to completely retrain to become accountants or administrative clerks. The CIT interviews suggest that the factors that push these decisions are not day-to-day infrastructural constraints *per se*—these are accepted by those who remain past the look-see—but infrastructural issues tipping into personal safety issues. Women, particularly in rural parts, most frequently described incidents where they felt especially vulnerable.

This may also explain the abundance of female worker departures in these settings: ‘The problem is on women, when they report here […] you will just find some files on your table, ‘I am married somewhere so I need a transfer to join my partner’. (CHMT, rural). One manager appeared suspicious of marriage as an exit point for quitting rural posts: ‘Others decide to get married, I don’t know if the time to get married has reached or they don’t want to stay in rural areas any longer’. (CHMT, rural).

Those particularly pushed to go, or even just moving on, had reportedly *noticed promises failed to materialise*, this was driven by wider issues related to staff shortages: ‘…when they start we are instructed that one must work for 3 years then you will go for further training, when the time comes, you find that your chances of attending training is low because you are alone at a facility and if you leave then the health facility is closed. You find that health workers are dissatisfied, so they quit’. (CHMT, urban).

Concurrently, nearly all rural district health officials (97 %), see Table 3, reported attempting to offset the drawbacks of the rural setting by offering access to training opportunities, but there were fewer reports from those representing the urban districts (56 %, *p* = 0.0001). To further offset rural settings, these district health officials were also more likely to report offering pension schemes (74 % compared to just one report from an urban representative).

**Table 3:** Differences in incentives reported by district health officials to be awarded in their respective districts

Different according to setting?		Setting *n* (%)		
Totals	Overall *n* = 43	Rural *n* = 34	Urban *n* = 9	*P* value
Salary top ups	10 (23)	8 (24)	2 (22)	0.934
Allowances	39 (91)	32 (94)	7 (78)	0.133
Accommodation	20 (47)	16 (47)	4 (44)	0.889
Access to training/education	*38 (88)*	*33 (97)*	*5 (56)*	*0.001*
Improved human resources management systems	18 (42)	15 (44)	3 (33)	0.56
Improved access to resources	16 (37)	12 (35)	4 (44)	0.614
Access to loans	29 (67)	24 (71)	5 (56)	0.392
Pension scheme	*26 (60)*	*25 (74)*	*1 (11)*	*0.001*
Meals for staff	3 (7)	3 (9)	0 (0)	0.356
Free uniforms	38 (88)	31 (91)	7 (78)	0.265

Statistically significant at conventional levels (*p* < 0.05)

Nevertheless, *poor management and communication between workers and their seniors*, such as heads of departments, was noted in both rural and urban settings as a key reason behind workers feeling pushed to leave. A rural manager had this opinion: ‘It is [mainly because of] bad relationships between workers and their leaders, a person can make mistakes but instead of calling her, discuss the matter and give her warning, they frustrate her by saying bad things and lies against her until she leaves…but later you come to find out that she was not a bad person’. (CHMT, rural).

Those feeling pushed to leave were similarly likely to *have had bad experiences with the community*: ‘One thing that affects motivation is particularly when the community which surrounds them doesn’t value their contribution, don’t appreciate their services, and so the health worker doesn’t feel that they are valued’. (CHMT, urban). Such accounts often involved workers being blamed for patient deaths, even though the big factor contributing to patient fatalities appeared to be that patients were regularly brought very late to the health facility, and so were in critical states on arrival.

On the flip side, for *The Stayers*, having a *good relationship with the local community* was mentioned as the most important motivating factor, described as indirectly *settling them into their posts*: ‘Others are just committed to serve and they say this is the place for me to work as long as the community appreciate the service they provide…therefore they will work wholeheartedly, they will not mind about distance, they will not mind about lack of mobile network, they will just work hard’. (CHMT, urban).

Across all these analyses we observed, overall, a general perception that many rural workers would readily leave for urban posts when these became available or after retraining, because these were seen to offer better incentives, infrastructure and wider prospects. Although the survey data suggest that some of the benefits of rural packages are better on the long term (e.g., pension schemes), not all incentives were from packages. Health workers in urban areas can get their extra income through locum work, or other paid opportunities which are more likely to be available in cities. As one manager put it: [workers in rural districts] they do not have the option of doing part time work’. (CHMT, rural).

c) What critical incidents do those who stay face?

**Fig. 4:**
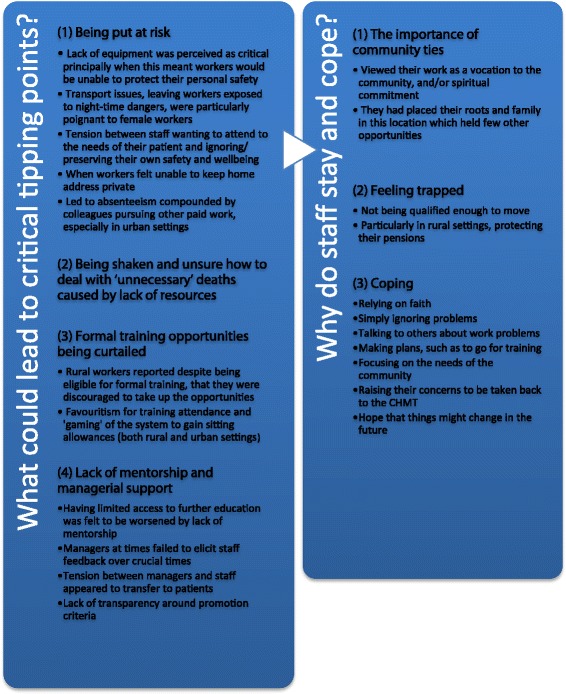
Contrasting critical tipping points with why healthcare workers say they stay

Healthcare workers raised general problems, and a few critical incidents that illustrated them in extreme case scenarios; these are summarised in the first column of Fig. 4.

Overall, from *The Stayers*’ perspectives, situations that were perceived as infrastructural were accepted, or overlooked. This was particularly true when respondents felt the situation was not specifically affecting them, but due to circumstances within the United Republic of Tanzania that affected most healthcare workers: ‘I have to accept the environment because whom could I blame? Medicines are not available…I just accepted the circumstances because this is vocational work and when you try to look around you do not see whom to blame, anyway you do not know where the problem started from’. (CIT, rural).

This culturally adopted ‘resigned fatalism’ is prominent in contexts in which people have had to become resilient and adaptive to system shortfalls, but were so pervasive that narratives exposing these constraints were at first easily overlooked. Nevertheless, sub-themes repeatedly emerged that challenged the acceptability of these shortfalls when they contributed to the staff themselves *being put at risk*. As such, *lack of equipment was perceived as critical*, *principally when this meant workers would be unable to protect their personal safety.* In the context of HIV, where gloves are often unavailable for months on end or else supplied in insufficient amounts, it was noted: ‘I get gloves disposable 100, surgical 40 in a month, so now do you think how many procedures am I going to perform? Because each procedure needs to wear new gloves, how many procedures will I perform?’ (CIT, rural).

*Transport issues*, *leaving workers exposed to night-time dangers*, *were particularly pertinent to female workers*: ‘I was called at 3 am in the night to attend a pregnant woman…I came with a kerosene lamp…don’t you see this is a very dangerous environment and I was just called on the phone? You come on foot without even a bicycle or a vehicle and this place is very dangerous’. (CIT, rural). Another female worker described having to use a ferry at night to transport patients to the nearest hospital. Many times she had to return alone in the boat with the driver. Narrating a critical incident she spoke about a time when the boat machine failed and they started to drift out to sea, making her fear for her life.

We observed an uncomfortable *tension between staff wanting to attend to the needs of their patient and ignoring/preserving their own safety and wellbeing*. Again, in relation to transport safety at night, nurses repeatedly described needing to accompany lone and fragile patients. The patient paid the fare out: ‘…but remember that it is midnight and you are alone…you have to beg the taxi driver to get you back because there is no return fare’. (CIT, urban).

Another concern seen to put staff at risk was *when workers felt unable to keep home address private*, and this was sometimes seen as more feasible if they lived away or in towns: ‘the story [in the village was] that a certain nurse was asked for help at night [these people] knocked on the door pretending asking help for a patient…Unfortunately those people were robbers […] she was beaten by them to death. Knowing of an occasion like that has truly affected me. I don’t know when I will shift from [the village] to town’. (CIT, rural).

These fraught circumstances, *led to absenteeism compounded by colleagues pursuing other paid work*, *especially in urban settings.* In turn, reductions in the number of staff, both on duty and at proximity for emergency callouts, compounded infrastructural limitations. Relatedly, when these constraints had fatal consequences for patients, they were harder to overlook. A few workers described *being shaken and unsure how to deal with ‘unnecessary’ deaths caused by lack of resources.* As one nurse put it: ‘…that could be a maternal death on the spot, you see it directly…you know if I fail to get something it means she can die at my side, while the possibility of assisting her is there…I think she could die because of a minor problem coming from the drip’. (CIT, rural). Handling these frustrations and emotions was never discussed.

A core theme in critical incident narratives formed around *formal training opportunities being curtailed*. What is interesting about this theme is how these situations were seen to manifest. In contrast to survey reports, *rural workers reported that despite being eligible for formal training*, *they were discouraged to take up the opportunities by their managers:* ‘He told me because I am working in the village so I don’t need higher education’. (CIT, rural). It appears these workers, unable to pay for their own studies, were tied too hard to fill mid-level rural posts, at the district’s convenience, rather than at their own—or indeed the system’s—benefit.

*Favouritism for training attendance and ‘gaming’ of the system to gain sitting allowances* was described by MLPs: ‘It is like confidential because someone will go to the seminar and she is ordered not to tell anyone else; if it is right why don’t you tell everyone that you have gone for a seminar? If what they are doing is right why not inform others?’ (CIT, urban). It was also revealed that at times senior staff members were going for trainings that were neither relevant to their role nor aimed at their specific specialities.

Relatedly, *lack of mentorship and managerial support* was described as one of the biggest issues faced by workers in both urban and rural settings. *Having limited access to further education was felt to be worsened by lack of mentorship*, as this experience illustrates: ‘Now you find yourself not able to go to school, you get a patient also you don’t have anyone to ask’. (CIT, rural). Negative leadership could also be a problem—‘at least their good words would motivate us, rather than always words of blame’. (CIT, rural).

Surprisingly, *managers at times failed to elicit staff feedback over crucial issues*, for instance this included managers moving workers to new posts, without them being consulted: ‘I have the right to get services, like to be informed about something, but my bosses have planned what to do to me and they told me suddenly’. (CIT, rural). Others reported being blamed for neglecting administration when they were actually being overworked and having to prioritize: ‘We didn’t write an evening report, because we were so busy. We didn’t even get the time to sit down, but we managed to attend patients well. When a thing like that happens, you find that the in-charge gets angry. To be honest that incident pained me because I didn’t do that on purpose. It was because of time and I was very tired’. (CIT, urban).

*Tensions with managers appeared to transfer to patients*, as this illustrates: ‘…because when you are having a lot of feelings in your mind you cannot properly attend to your to patient, you might think that they are the cause of all your problems, since you feel totally discouraged. You could even say unpleasant words to one who is innocent’. (CIT, rural).

*Lack of transparency around promotion criteria* was perceived as a critical issue, particularly by MLPs, as one interviewee put it: ‘It’s about ten years now since I got promoted, I believe my salary rank should be much higher, due to my age at work and my job title now…but I am not an expert on analysis of these employment claims…it needs someone who is transparent and who can explain well to you that you deserve this promotion—to get it [promoted], you need someone who can give you explanations: it is done this way and that way. So that you understand what is needed, and if you even deserve to be in a position or rank that is not now yours’. (CIT, rural).

### 2. Exploring retention and positive coping

d) Why do staff stay and cope?

See summary in second column of in Fig. 4. Echoing managers words about *the importance of community ties*, *The Stayers* reported being settled to their posts often in the face of harsh working environments, because they *viewed their work as a vocation to the community*, *and/or spiritual commitment.* ‘I dedicated my heart to this work with my faith, and I ask myself if I go out of this place who else will come to work here?’ (CIT, rural). Or correspondingly, because *they had placed their roots and family in this location which held few other opportunities*, for instance: ‘Due to the time I have spent here, I don’t think of shifting; it will distort everything; it will also bring confusion to my children’. (CIT, rural).

Many in this position also paradoxically spoke about *feeling trapped*, *not being qualified enough to move*, although: ‘If it is possible to move I would, unfortunately I don’t have such education,’ (CIT, rural), ‘I tried to search for another job without success, it is better to stay here’. (CIT, urban). Or *particularly in rural settings protecting their pensions:* ‘I continue working here to protect my pension’. (CIT, rural).

Healthcare workers were asked to describe *coping* with the issues that had caused them to be demotivated or consider leaving their job. Respondents often talked about *relying on faith*, making statements such as the commonly used phrase: ‘I leave everything to God’. Another common strategy was *simply ignoring problems*. More proactive approaches were rarer, but included *talking to others about work problems*, many MLPs for example asked each others’ views before deciding on a course of action. *Making plans*, *such as to go for training*, and *focusing on the needs of the community* were also reported to help overburdened workers cope.

One respondent revealed that the CHMT conduct monthly visits to their facility so this provides an opportunity for staff to report their problems to the Council. After taking part in the CIT interviews, some study participants did *raise their concerns to be taken back to the CHMT*. They also revealed that they were given a number of promises and *hope that things might change in the future*: ‘They told us to wait, things will be changed, so here we are waiting while we continue with working’. (CIT, urban).

## Discussion

These analyses confirm many of the factors that are already known from other Sub-Saharan African contexts about the challenges of rural recruitment and retention, but they also add a new layer of understanding: explaining them. This paper moves beyond reiterating the familiar woes of under-resourcing that hamper efforts to ensure more equitable access to HRH and starts to draw out more nuanced details that can be used to inform effective and targeted policy efforts.

### Problems with recruitment and retention according to cadres

Mid-level cadres were especially problematic to retain and caused the most disruptions to continuity of care when they left. Our findings confirm the widespread use of MLPs in the United Republic of Tanzaniaa, as in other Sub-Saharan African countries [15–17], forming the backbone of the health service, particularly in rural areas. Moreover, in the United Republic of Tanzania, shortages of the highly skilled clinical workers not only a rural issue, but as a national one, similarly proportioned, in either setting; yet these shortages, likely due to multiple compounding frustrations and lack of mid-level workers, were more deeply felt in rural settings.

### Ways of leaving

These were identified as two types (*Look, See and Go* and *Movers On* leavers), and a group who remain in post (*The Stayers*) gives us deeper insights into why some staff leave, while others remain in post. Some *Look, See and Go* staff were described by CHMT members as unable to adapt to their environment. This was predominantly due to unfamiliarity with rural areas and the attendant poor living conditions or lack of acceptable housing, and unpaid salary or allowances.

A recent MoH report (2014–2019 projections) [18] estimates that the United Republic of Tanzania is not only facing an estimated 56 % of health care staff shortages but that they are also unevenly distributed. Councils complain that the current system of advertisements for recruiting health staff provides a chance for potential recruits to choose three regions they would like to be posted to. Consequently, rural and hard to reach places are less selected. They confirm, as we have found, that not all of the posted employees report. Others report to postings but quit soon after. Out of 4812 permits, which were utilised, a reported 63 % went to their respective stations, 13 % of these left for several reasons, often cited were delays in being entered into the payroll and receive salary payment on time.

The phenomenon of *Look, See and Go* is of particular interest because it highlights an area of recruitment policy and practice that could readily be improved. The practice of allowing health workers to line up a number of options, then ‘hedge their bets’ to choose the best one, is a waste of already scarce resources and could be addressed. Issues relating to inadequacies of government postings and transfer (P&T) procedures in the United Republic of Tanzania have recently been highlighted [19, 20].

Schaaf et al. (2013) refer to P&T as “mission inconsistent (MI)’, meaning that they are not conducted in a way that maximizes health outcomes or that respects the norms of health care worker professionalism’ (p. 1) [19]. Elsewhere (2015) [20], these authors note P&T to remain a ‘vexed and unresolved issue’ in many middle- and low-income settings, arguing that above all, political commitment to improving such practices and to new solution focused inter-disciplinary thinking and research is required.

### Reasons for wanting to leave

These were manifold and—as illustrated above—clearly tied to types of leavers. Nevertheless, commonalities related to three main areas: workplace and personal safety, training, poor management.

In neighbouring Malawi, staff shortages and overwhelming workloads have been shown to be major factors in pushing health workers to seriously contemplate leaving their jobs [21]. Previous research [22] has noted that aspects of infrastructure and poor management explained much of the variation in satisfaction in the United Republic of Tanzania. However, while Tanzanian workers flagged similar shortcomings, there was also a pervasive fatalistic acceptance of infrastructural limitations. Actually, what made them feel pushed to go most was when they experienced these limitations as direct threat to their own or patients’ safety. Indeed, unsafe handling of blood, housing and transport arrangements (in particular for female staff member, and those in rural districts) appeared to the biggest push factors. Echoing this, women working in a church-run rural Tanzanian hospital reported appreciating the safety of staying within the hospital compound [23].

Unsupportive or poor management was encountered not simply as passively failing to provide leadership and opportunity but also actively allowing unacceptable events to manifest, for example in situations where personal vendettas caused unfounded accusations against professional competencies. Such issues occur in many contexts and should be mediated by higher-level human resource management (HRM) intervention, which should buffer and protect the injured party. As for lack of opportunity, existing study in Tanzania similarly cite managerial favouritism from allocation of allowances, to who gets to attend further training or refresher courses [24].

In Uganda, it has been shown that training is sometimes not seen as a capacity building process, but rather as a way to get cash stipends, paid as allowances for attending [25]. Equally, the selection of those who attend these workshops was questionable. Since unqualified people were selected to attend workshops, which were not aligned to their expertise, therefore the person would not be able to benefit the organization in return and would share nothing of the experience.

We heard similar accounts in the United Republic of Tanzania, both our quantitative and qualitative findings have shown unfairness or lack of transparency in access to training and professional developments. These were critical factors that caused *The Movers on* to consider leaving. Other studies in the United Republic of Tanzania [10] revealed that health workers expressed strong feelings about limited training opportunities in rural areas. In our data, additional issues which rural workers faced were lack of replacement staffing to cover their training absences, as much as lack of opportunity. Urban workers were seen as having the opportunity to earn more money and save, and more able to invest in their own training to get themselves ahead.

### Resounding reason to stay

*The Stayers* resoundingly talked about having a strong sense of place and motivation to serve it; these workers needed community approval and described having their families and roots embedded in it. Similarly, previous research shows that female staff members tended to have their workplace determined by where their husbands were and to want to live close to their extended family and elderly parents, siblings or other relatives [23]. In such cases, the workers choose the community first, the job indirectly.

Practically, rural workers talked about feel tied by the promise of pensions. In sum, those that were able to settle, and had ties, stayed longer. For example, in Zambia, settling in newcomers, faced with the challenges of working and settling in rural settings after completing training in urban training schools, was seen as crucial when discussing health worker retention schemes [26]. Studies from Ethiopia and Rwanda recommend a focus on reducing turnover, encouraging the stability and motivation that is associated with a longer stay, and older workers [27].

## Conclusions

With respect to P&T, to avoid early *Look, See and Go* departures and hedging bets with applications, or ‘gaming’ postings, we recommend a centralised and monitored deployment system that matches worker profiles to the most suitable post for them. For example, the Senegalese Ministry of Health demonstrated some success in addressing geographical disparities in health worker density by introducing a temporary contracting system. The traditional system recruited health staff as civil servants, deploying them on an annual quota; these workers did not know where they would be going nor for how long. The new, flexible system recruited staff for a specific post, in a known location, for a specified amount of time, and this coupled with good housing and incentives, in the short term at least, appeared to work well [28].

Moreover, there is growing evidence of the utility in matching a candidate’s own background to the setting in which they are posted, with staff who grew up in a rural environment more likely to assume duties in these settings [27, 29]. Thoughtful recruitment policies should be able to prepare the right candidates for the right placements. Active integration on arrival from management, including ensuring settlement allowances are paid, management accompanying new staff to their location and introducing them to their communities, and even giving free mobile phone airtime, were all shown to help staff settle [30]. The practice of posting workers at the convenience of the district and simply filling staffing quotas in ‘hardship’ locations without a training agenda resulted in a notable paradox: neither fulfilling peoples’ potentials nor ultimately acquiring much-needed high calibre skill sets.

One solution to this training vacuum is seconding urban workers temporarily to enable the training of rural workers, either to cover or to run training on site for their rural counterparts. Training must be tackled by having guidelines and policies, available to all staff, that clearly outline, and are set up to help enforce, their entitlements, but crucially must be tracked and equitably awarded. Training should also be focused on making managers better, rather than simply aiming to improve clinical skill sets. In addition, a centralised system can also help institute greater transparency and hold managers accountable; tracking recruits’ progress and performance, incentives (allowances, training) over time; since all these areas were noted to lack follow-up.

Since not all infrastructural limitations can be tackled at once, we recommend an explicit focus on workplace and personal safety, fully prioritizing these and changing the organisational outlook that safety outside the hospital walls are not important. Certain issues such as safe handling of blood, night transport, and safe housing were persistently noted. Moreover, given the centrality of the community to health workers motivation to stay in post, a more interactive health services and community self-management model may improve workers relationships with community members.

For example, a focus on explaining the dangers of late presenting, which could be easily communicated in health centre poster messaging, or community billboards, may protect workers from shouldering the blame for poor health outcomes resulting from this. Community involvement in health services provision thus promises multifaceted benefits; including encouraging the community to take responsibility for help-seeking as well as sensitising them to health workers day-to-day working constraints.
